# Fatigue Damage of Aluminum Alloy Overhead Line Conductors Initiated by Fretting

**DOI:** 10.3390/ma18174103

**Published:** 2025-09-01

**Authors:** Andrzej Nowak, Paweł Strzępek, Piotr Korczak

**Affiliations:** 1 Faculty of Non-Ferrous Metals, AGH University of Krakow, 30-059 Krakow, Poland; annowak@agh.edu.pl; 2 Łukasiewicz Research Network—Institute of Non-Ferrous Metals, 32-050 Skawina, Poland; piotr.korczak@imn.lukasiewicz.gov.pl

**Keywords:** fatigue failure, fatigue damage, fretting, overhead line conductors, aluminum alloys, non-ferrous metals

## Abstract

Fatigue failure of overhead line conductors made of AlMgSi alloys is much more complex than fatigue failure of a single wire. The main difference lies in the fretting phenomenon, which is a significant mechanism initiating fatigue damage. It is generated because of micro-movements between individual wires or outer wires and overhead line fittings. Such movements are mainly caused by aeolian vibrations, which lead to degradation of wire surface, initiation of microcracks, and premature failure of multiple wires. Research based on laboratory experiments and modeling studies simulating real operating conditions made it possible not only to identify the mechanisms leading to failure but also to assess the impact of working conditions on their evolution. According to the obtained results, properly selected heat treatment parameters influence both the mass decrease of the wires and number of cycles to failure due to fretting fatigue. Further development of materials, protective coatings, and methods of durability prediction will reduce the impact of fretting on fatigue failure and thus increase the reliability of power lines.

## 1. Introduction

The demand for electrical energy is projected to increase by 50–100% in the European Union in the next 25 years [1,2,3], and thus it requires constant development of power grids both in terms of energy sources as well as overhead line fittings and conductors [4,5,6,7]. Considering metals that can be used for transferring electrical current, copper is one of the best electrical conductors in ambient temperature, being only second to silver [8,9,10]. Even the generally accepted unit for describing the electrical conductivity of metals uses copper as the point of reference, where 100% is the value of electrical conductivity of copper in the annealed state (IACS—International Annealed Copper Standard), which was established back in the 1914 [11]. However, due to the high prices of these metals [12,13], silver is not used for wiring, and the use of copper is limited to applications that require a low volume of the material or have very high conductivity. Since the density of aluminum is more than three times lower than that of copper, its electrical conductivity is around 60% IACS, and historically its price has always been several times lower than that of copper, it is an obvious choice for long-distance overhead line conductors [12,14,15,16]. On the other hand, the tensile strength of pure aluminum is not sufficient to sustain the weight of conductors for long periods of time [17,18,19]. Therefore, overhead lines are usually constructed using ACSR (aluminum conductor steel reinforced) conductors, with a steel core (usually 7 wires) and multiple layers of aluminum wires or AAACs (all aluminum alloy conductors), which use only AlMgSi wires [20,21,22].

**Figure 1:**
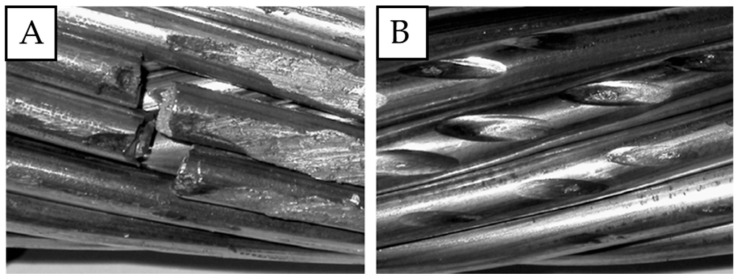
Examples of fretting on the wires subjected to cyclic fatigue tests (number of cycles to failure 6.4 × 10^6^, amplitude 0.9 mm); fatigue failure (**A**) and damaged wires (**B**); own research [20].

However, high tensile strength alone is not enough to provide long-term and failure-free operation time. Apart from that, particularly important is the fatigue resistance of aluminum wires, which is influenced by many factors, for instance fretting fatigue [23,24,25,26,27]. The fretting phenomenon is produced by the combination of cyclic loads and micro-movements between the contact surfaces of individual wires or outer wires and overhead line fittings. It leads to the degradation of the surface layer and initiation of microcracks, and as a result, a premature failure of the conductor [28,29]. To prevent such failures, AAAC rather than ACSR or aluminum conductors are preferable, as they are characterized by a favorable ratio of electrical conductivity and mechanical strength (unlike ACSR, in the case of AAAC, the whole cross-section is conductive) [20,30,31]. Overhead line conductors are widely used in Europe, and thus research on their durability and fatigue resistance are of the most importance [20,22,29,30]. Examples of damage originating from fretting are presented in Figure 1.

The phenomenon of fretting is the subject of study using experimental modeling research on brand new conductors, experimental research on conductors at the end of operation life or due to a failure, and finite element method (FEM) simulations. Omrani et al. assessed the fretting fatigue resistance of overhead conductors using a numerical model and test verification. They claim that the contact force studied through uniaxial loading tests has an impact on the fretting fatigue life for lower alternating stress amplitudes [25]. In their work [32], Poon et al. studied fretting of multi-wire copper conductors used as submarine power cables. Using FEM modeling, they were able to identify the fretting fatigue relationships between representative local 2D and 3D modeling via matching contact pressure, and global multi-wire contacts. With the help of their model, they were able to predict fretting fatigue resistance and proved that increasing lay angle, contact size, wire diameter, and friction lead to its reduction. In their other works, Poon et al. [33,34] have analyzed submarine cables including four varied approaches; i.e., a coupled aero-hydroelastic dynamic simulation framework for global dynamic analysis; simplified full cross-section sub-element modeling of a submarine power cable; a local submarine power cable inter-wire conductor frictional contact model; and local, high-resolution, representative cylinder-on-flat modeling for multiaxial fretting fatigue and wear simulation. Another work using FEM simulations was published by Wang et al. [35], where apart from fretting, they considered corrosion effects. They provided results showing that the increase of contact force from 120 N to 240 N reduces the fretting fatigue resistance by 25%. What is more, when the corrosion effect was added, the damaged zones formed after less than half of the cycles needed when no corrosion environment was applied. Thomas et al. in their study [36] considered the influence of internal stress states inside of the wires. This helped to develop fretting fatigue criterion, and thus the prediction model, which provides probable locations of fretting fatigue failure of the overhead line conductors. Overall, most of the papers on fretting fatigue resistance are focused on the area near the suspension clamps, as this is where the most damage takes place due to fretting [37]. Many techniques and methods of investigating the fretting fatigue cracks are presented and collectively described in the review papers published by Kong et al. and Kaimkuriya et al. [38,39]. Their papers also discuss the influence of hardness, temperature, and residual stress. The key conclusion of [39] is that designers must take these factors into account, especially when high-cycle fatigue applications are in question. They observed that high temperature significantly reduces the fatigue life of components with low thermal conductivity. What is more, exceptional hardness was found to be the origin of earlier fatigue failure due to lower ductility. Ding et al. [27] and Zhu and Chen [40] have experimentally verified the FEM analysis results of bending fretting damage of AlMgSi materials. According to their results, the fatigue lifespan of AlMgSi alloy decreases significantly with the increase in peak bending force under the same normal load. Garcia et al. in their experimental paper [41] conducted fretting fatigue tests at ambient temperature and at 75 °C, since this is the usual operating temperature of overhead conductors for transmission lines made of AlMgSi alloys. They concluded that the wires tested at the increased temperature had their fatigue life reduced by up to 72% in comparison to the wires tested at the ambient temperature. Apart from methods for predicting where and when the fretting damage will take place, researchers have worked on methods of preventing the fretting damage and fatigue cracks, starting with the proper materials selection, modifications of construction (various segmental conductors), and application of protective coatings [22,36]. Considering that local damage resulting from fretting may cause failure of the whole conductor, and therefore a serious breakdown of certain segments of the power grid, it is important to find ways to prevent it. Thus, the aim of the current paper was to verify the effect of the heat treatment of individual wires on the fretting fatigue resistance of the entire overhead line conductor. The results might suggest the prospective direction of development preventing the risk of failures.

## 2. Mechanism of Fretting Formation

**Figure 2:**
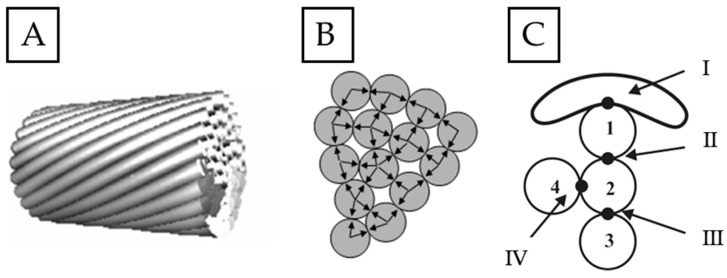
General view of an AAAC overhead line conductor (**A**), with marked interactions between individual wires (**B**), and spots susceptible to fretting damage (**C**); I—the point of contact between the wire (1) of the outer layer and the overhead line fitting; II and III—the points of contact between the wires (1–3) of different layers; IV—the point of contact between the wires (2, 4) of the same layer; own research [20].

The phenomenon of fretting in the case of overhead line conductors is complex as it is the result of the interaction between many factors [20,42]. The most basic mechanism covers the sliding or oscillating movement between the adjacent wires’ surfaces under the influence of cyclic loads, as presented in Figure 2 [20,24,27]. Such micromovements, although very small (several micrometers), generate contact stress and local frictional wear [20,29,43].

**Figure 3:**
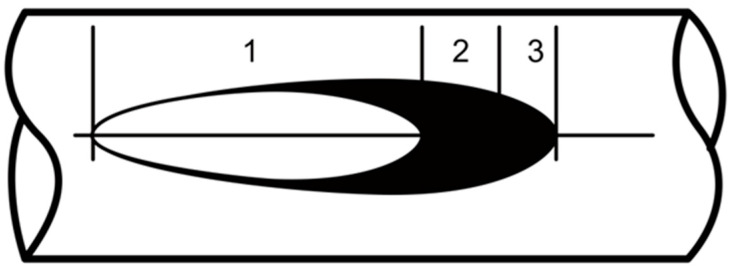
Typical elliptical fretting damage: non-slide zone (1); micro-slide threshold zone (2); accumulation of the fretting products (3); own research [20].

As a result of said friction, surficial fretting damage occurs in the form of scratches, dents, seizures or even detachments of the outer layer. Corrosive processes may occur at the spots where the formation of fretting damage is probable due to exposure to changing weather conditions (moisture from rain, snow, or fog; drastic changes of the temperature; aggressive environmental pollutants) [20,44]. Local stress concentrations that are formed in the contact spots (Figure 2C I–IV) favor the initiation of microcracks leading to wire failure, and consequently conductor failure, as presented in Figure 3 [20,23,25].

**Figure 4:**
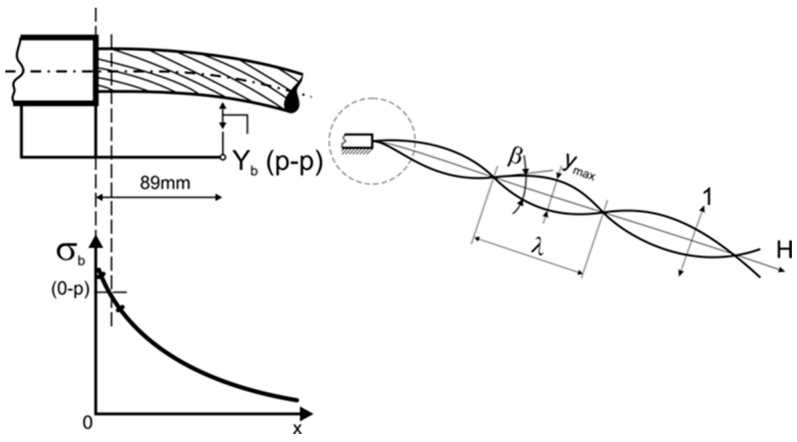
The influence of aeolian vibrations on an overhead line conductor near the clamp; local stress in the area near the clamp (σ_b_); peak-to-peak displacement at 89 mm (the most vulnerable to fretting damage area) from the conductor clamp (Y_b_(p-p)); the angle between the vibration wave and the conductor axis (β); the maximum amplitude of the vibration at a given point of the conductor (Y_max_); the wavelength of the vibration as the distance between two consecutive points in the same phase of the wave (λ); the axis of the conductor (H); main direction of the wind (1); own research [20].

One of the key factors generating micromovements leading to fretting damage is aeolian vibrations of the overhead line conductor [20,29,42]. They are generated by low-speed winds, causing high-frequency, low-amplitude oscillations of the conductors. Long-term exposure to such vibrations may cause fretting damage leading to fatigue failure, as presented in Figure 4 [20,22,42].

Aeolian vibrations accelerate surface wear and initiation of fretting damage by forcing micromovements between individual wires of the conductor [28,29]. This results in the reduction in the durability of the conductor and thus the risk of premature failure [20,22]. There are of course other probable causes of fretting damage, including mechanical or electrochemical aspects [27,29]; however, since these problems are not raised in this paper they will not be discussed further.

## 3. Materials and Methods

As already mentioned, research on fretting fatigue is conducted using test stands reflecting real operating conditions of conductors and/or numerical simulations [20,22,25,32,38]. In the current study, the test materials were wires made of typical AlMgSi alloy for electrical applications; i.e., AA6101 alloy in the T4 temper (solution heat-treated and naturally aged to a substantially stable condition). The chemical composition of the material was verified using an optical emission spectrometer Foundry-Master Xpert (Oxford Instruments, Abingdon, UK). The diameter of the individual wire of the overhead line conductors depends on the application and is typically between 2 mm and 5 mm [45], therefore for the tested wires, the chosen diameter was 3.12 mm. Three variants of heat treatment were selected for the study of fretting resistance:

- Wires with no artificial aging;
- Wires subjected to artificial aging for 2 h in 160 °C;
- Wires subjected to artificial aging for 9 h in 160 °C.

Times of the artificial aging were selected based on the Vickers hardness results conducted on the cross-section of the wires with HV1 load using TUKON 2500 hardness tester (Buehler, Lake Bluff, IL, USA). The tester had a test load accuracy of measurement of ±1% and the accuracy of the indentation diagonal was 0.02 mm. Six indentations were made at an ambient temperature on the wires subjected to heat treatment in the range of 1–12 h (samples were removed every hour).

**Figure 5:**
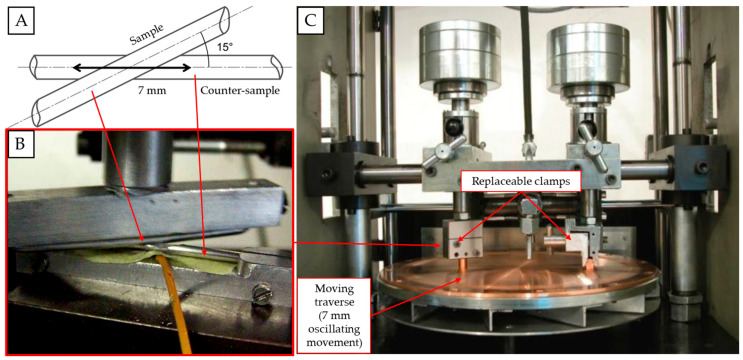
Schematic view of the conducted experiment (**A**); photograph of the mounted sample and counter-sample (**B**); general view of the test stand (**C**), own research.

In order to simulate the fretting phenomenon, a special mount for samples was designed ad hoc and adjusted to the existing test stand used to verify the abrasion of wires (Raqun sp. z o. o., Gorlice, Poland). According to the international standard [45], the average angle between consecutive layers of the conductor is 15°. Therefore, sample and counter-sample were set at that angle to best reflect real operating conditions. The surfaces of both sample and counter-sample were cleaned using acetone and dried with a clean cloth prior to mounting them. The counter-sample was installed parallel to the movement axis, and the movement amplitude was set at 7 mm. The contact force was simulated with a load of 2 N and 50 N. The load values were chosen to best reflect the lowest fretting load that can cause damage, and the typical fretting load that can be recorded during operating life of an overhead line conductor [46] in the areas of contact marked in Figure 2. Such loads are generated by the tension load of the conductor, its geometry, and the way it is mounted and is affected by the fitting of the line, as marked in Figure 4. In order to properly verify the influence of the fretting damage on the fatigue resistance of wires, three samples were subjected to tests in each condition, and the results are presented as average values. The calculated means were analyzed using a single-factor ANOVA (analysis of variance) test. The diameter of the tested samples was 3.12 mm, and their length was 00 mm, as this was the length needed for further fatigue tests. The experiment is schematically presented along with the test stand in Figure 5. The tests were conducted at an ambient temperature.

After specific number of cycles (up to 10,000), the samples were disassembled to verify their mass decrease using the RADWAG AS.R2 laboratory scale (Radwag Wagi Elektroniczne, Radom, Poland) with a scale interval of 0.0001 g in an air-conditioned laboratory at a constant, measured temperature. Samples were also subjected to scanning electron microscopy (SEM) analysis using a Hitachi S-3400N microscope (Hitachi Ltd., Tokyo, Japan) with the accelerating voltage set at 15 kV, and various magnifications.

**Figure 6:**
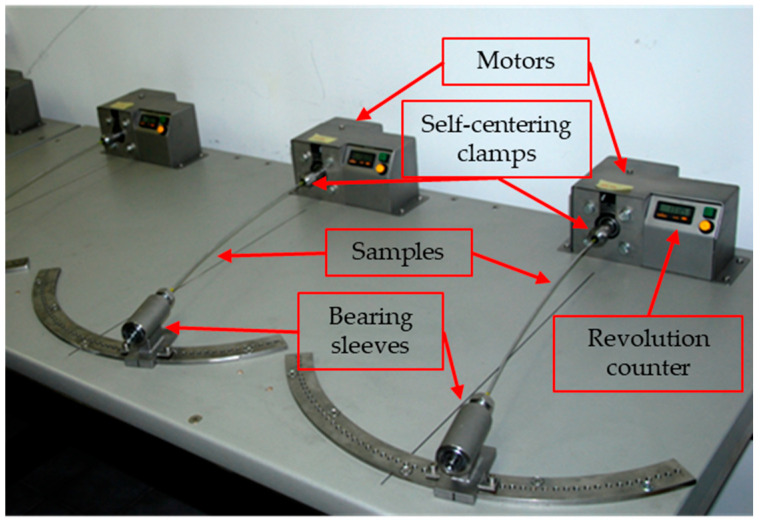
Test stands used during the cyclic rotation bending tests.

When the maximum number of fretting cycles was applied, the wire samples were subjected to fatigue strength tests using a device built ad hoc for the cyclic rotational bending test, which allowed for wire testing with various cyclic loads, as presented in Figure 6. The number of cycles and the applied load was monitored throughout the test. The tests were conducted at an ambient temperature.

The length of the tested samples was the same as in the case of fretting tests; i.e., 400 mm, and their diameter was 3.12 mm. By properly applied deflection of the sample, it was possible to obtain a desired value of stress in the fatigue testing, as presented in Table 1 [16,20,21,22].

In order to determine the Wöhler’s curves for each of the individual variants, three samples were investigated in each of the amplitude stress tests. The calculated means were analyzed using a single-factor ANOVA test. The experimental research was conducted until failure of samples both for samples damaged by fretting and samples that were not affected by it, to create the point of reference. The frequency of movement expressed by the rotational speed was 3000 rpm.

## 4. Results and Discussion

The results of the chemical composition analysis are presented in Table 2 along with the standardized elements amounts for the AA6101 alloy [47]. As presented, the measured results fall within the standardized values, proving that the tested materials were obtained from the desired alloy.

**Figure 7:**
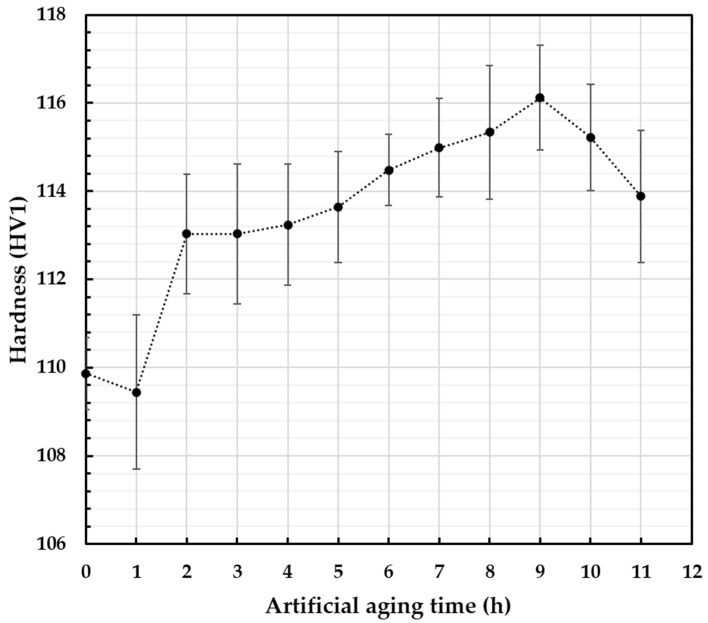
Vickers hardness results conducted on the cross-section of AA6101 wires subjected to artificial aging at 160 °C.

Prior to fretting tests, proper heat treatment was applied. To determine the most preferable times of aging at 160 °C, samples were subjected to Vickers hardness analysis. The results are presented in Figure 7. Based on the presented results, it can be stated that up to 2 h the aging time was not long enough to activate precipitation hardening. The material softened slightly after 1 h of heat treatment. The hardening effect could be observed starting from 2 h up to 9 h of heat treatment. After that time, overaging was observed and the recorded hardness started to decrease. This corresponds well with the fact that increasing aging time up to around 10 h increases hardness of the AA6101 alloy [48,49], and that higher hardness is generally associated with higher abrasion resistance [50,51,52]. Therefore, for further analysis, the artificial aging time was selected as 2 h (the time needed to activate the precipitation hardening) and 9 h (recorded peak of hardness).

**Figure 8:**
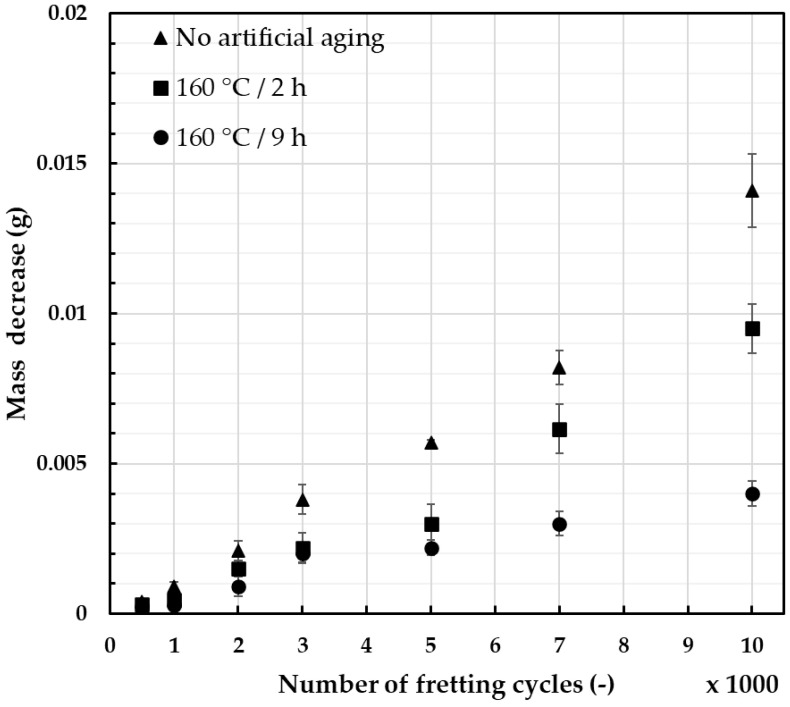
Mass decrease during simulated fretting cycles of AlMgSi wires; the diameter of wires was 3.12 mm, the load was 2 N, and the amplitude of movement was 7 mm.

**Figure 9:**
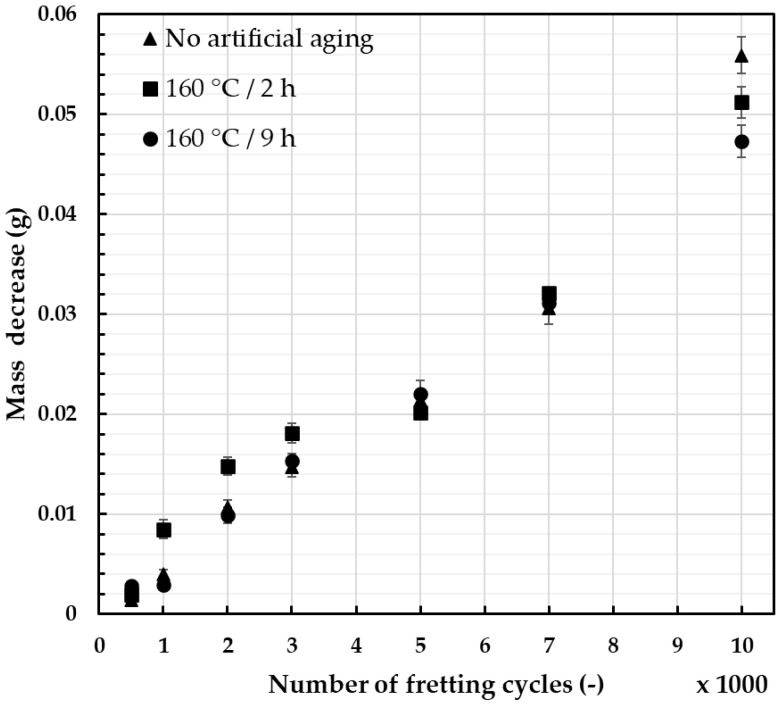
Mass decrease during simulated fretting cycles of AlMgSi wires; the diameter of wires was 3.12 mm, the load was 50 N, the amplitude of movement was 7 mm.

The conducted experiments made it possible to assess the influence of stress, amplitude, micromovement frequency, and environmental conditions on the failure mechanisms during fatigue strength tests. Such experimental simulations allowed the comparison of heat treatment parameters on the susceptibility of materials to fretting damage and its effects. The relation between the mass decrease and the number of fretting cycles with identical load and movement amplitude is presented in Figure 8 (2 N load) and Figure 9 (50 N load), with error bars providing information on the standard deviation of the recorded values.

For the assessment of wear due to the fretting damage, the mass decrease was chosen due to the simplicity and repeatability of the measurements. When a lower fretting load was applied (Figure 8), the increases in the mass decrease were quasi-linear and showed how much the heat treatment can affect the recorded values. However, the typical fretting load values recorded during operating life of the overhead line conductor were closer to 50 N [46], and these results provide more adequate information. As presented in Figure 9, the differences in measured values seemed to be insignificant. Wire subjected to 2 h of artificial aging at 160 °C showed the influence of geometry of the contact surface on the level of wear. Its geometry reached the maximum contact area after about 5000 cycles, leading to a significant reduction in unit pressure. On the other hand, the wire artificially aged for 9 h showed the lowest mass decrease, especially after a higher number of cycles. This corresponds well with the obtained hardness results and the fact that higher abrasion resistance is generally linked to higher hardness of the material [50,51,52]. The calculated average values were analyzed using ANOVA testing and the results are presented in Table 3. Based on the calculated *p*-values, it can be determined that when the load was 2 N, the values showed much more difference than when the load was 50 N.

**Figure 10:**
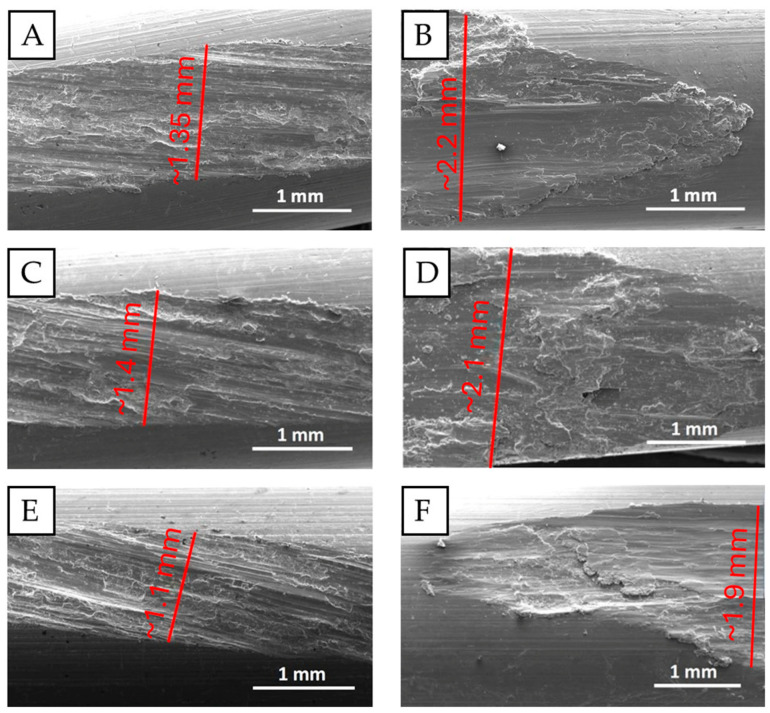
The width of the fretting damage trace after 10,000 fretting cycles; 2 N load (**A**,**C**,**E**); 50 N load (**B**,**D**,**F**); wires with no artificial aging (**A**,**B**); wires after 160 °C/2 h artificial aging (**C**,**D**); wires after 160 °C/9 h artificial aging (**E**,**F**).

The samples after simulated fretting damage were analyzed using SEM, which provides the possibility to identify characteristic surface damage in the contact zones of the wires, and thus precisely determine the geometry of the worn surfaces [20,23,24,25,26,27,28,29]. By analyzing the width of the fretting damage trace presented in Figure 10, it is possible to notice the influence of the load and the heat treatment on the wires’ resistance to fretting. The size of the fretting trace was measured in the widest damage area.

In real operation conditions for conductors, the contact force varies and depends on operating conditions (such as vibrations, humidity, contamination, temperature) as well as the structure of the conductor, the placing of the wire in the conductor, contact with overhead line fittings, and the distance from the clamp [22]. In each of the tested cases, the nature of the fretting damage changed as the load increased. As presented in Figure 10, when the load was only 2 N, the fretting damage showed mainly as a slipping effect, and its surface was evenly worn, regardless of the applied heat treatment. The entire fretting trace was of similar width in each case. On the other hand, when a 50 N load was applied, the fretting damage was much wider and more diverse. The damage was not homogeneous in width. There were visible slipping zones but also large amounts of deformed and sticking material, which were the products of abrasion. The width of the damage was the narrowest when 9 h of heat treatment was applied, which confirms the measurements of the mass decrease, and the fact that as the artificial aging time increases so does the hardness, and thus the abrasion resistance [48,49,50,51,52].

**Figure 11:**
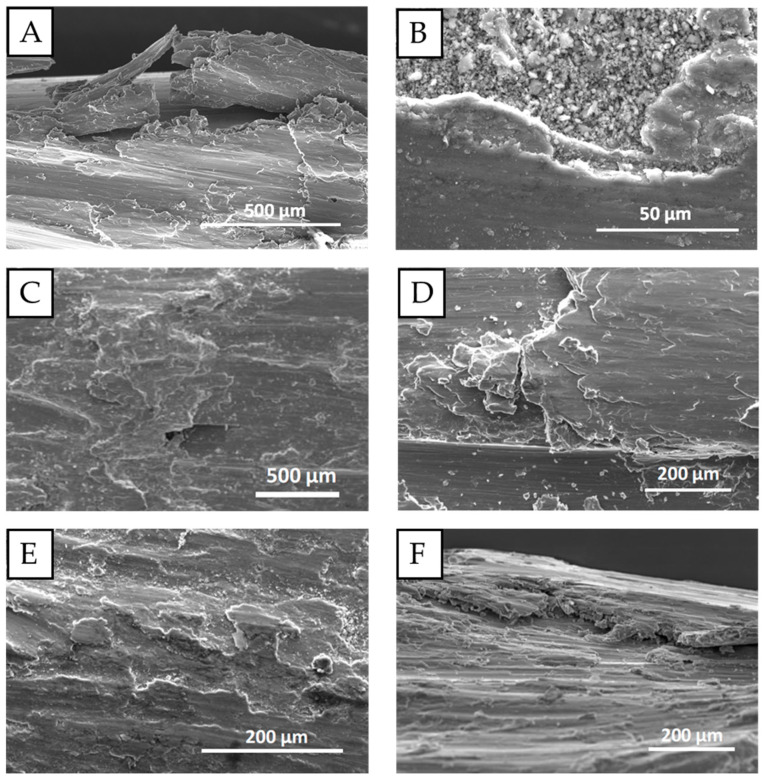
Surficial microdamage in the areas affected by fretting; the load in all cases was 50 N; wires with no artificial aging (**A**,**B**); wires after 160 °C/2 h artificial aging (**C**,**D**); wires after 160 °C/9 h artificial aging (**E**,**F**).

Further analyses of the surface damaged by fretting showed the presence of microcracks, delamination, and adhesive material. Al_2_O_3_ particles, which may accelerate the surface degradation, were also observed. Examples of such damaged surfaces due to fretting are presented in Figure 11 in various magnifications. Surface damaged to such an extent may initiate fatigue failure as it is generally known that regions with the highest surface roughness and stress concentrations are the origins of fatigue damage [20,22,26]. The presented images are excellent examples of stress concentrators leading to the development of said fatigue failure [23,24,25]. However, judging by the images, it seems that the samples after heat treatment had less developed surficial damage and did not suffer from such extensive delamination as the wire with no artificial aging.

**Figure 12:**
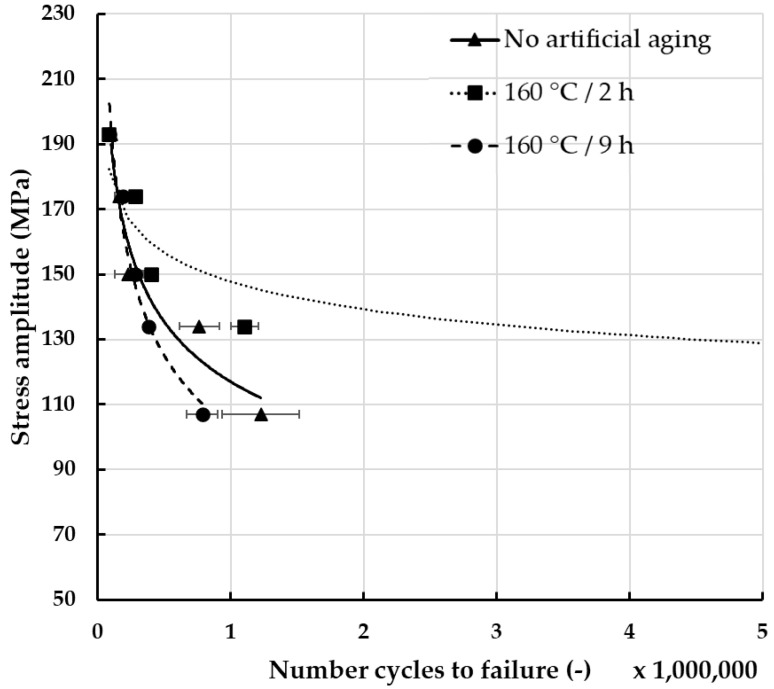
Fatigue strength of AlMgSi wires subjected to various artificial aging conditions.

In order to assess the impact of fretting damage on the fatigue strength of the wires, it was necessary to evaluate it first regarding wires that were not subjected to fretting, to create a point of reference. The results of these experiments are presented in Figure 12. Analysis of the measurements showed noticeable differences between the tested materials. The highest fatigue resistance, regardless of the applied stress, was recorded for the wires after artificial aging at 160 °C for 2 h. What is more, regarding lower values of stress, the wires were characterized by the so-called unlimited fatigue strength [22]. This can be attributed to the fact that artificial aging improved the mechanical properties and thus the fatigue strength [16,53,54]. On the other hand, the lowest fatigue strength was recorded for wires after artificial aging at 160 °C for 9 h. This is surprising, considering that wires after heat treatment in these conditions exhibited the lowest mass decrease. Rocha et al. attribute lower fatigue strength of materials with higher mechanical properties to higher residual stresses and very high yield stress [55].

**Figure 13:**
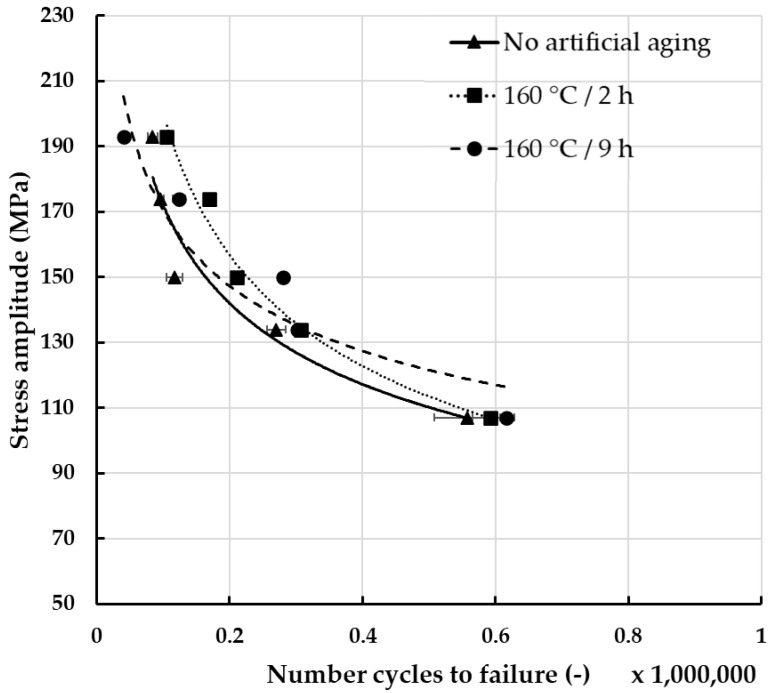
Fatigue strength of AlMgSi wires subjected to various artificial aging conditions and fretting damage with the load of 50 N.

Knowing the fatigue strength of the non-damaged wires, it was possible to compare them with wires subjected to fretting damage initiated by the load of 50 N. The results of fatigue strength of damaged wires are presented in Figure 13. A significant decrease in fatigue resistance was noticeable due to the location of stresses within the fretting damage. When compared to the reference results presented in Figure 12, it can be stated that fretting had the greatest impact on the fatigue strength of wires subjected to artificial aging at 160 °C for 2 h, as it decreased from unlimited resistance to almost 600,000 cycles to failure at lower stress amplitudes. Regarding wires with no artificial aging, their fatigue strength decreased by approximately half. The lowest decrease was recorded in the case of wires subjected to artificial aging at 160 °C for 9 h. When their surface was not damaged, their fatigue strength was the lowest, and after fretting it was the highest when the stress amplitude was under 150 MPa. Overall, the differences between individual materials decreased and the fatigue resistance of the tested wires was between 100,000 and 560,000–620,000 cycles, depending on the applied stress and heat treatment conditions. Slightly lower values were recorded by Sadeler et al., but in their research the load of fretting was two times higher [56]. However, researchers generally agree that fretting reduces fatigue life dramatically due to the introduction of shear stress on the surface through the contact between two wires [20,23,24,25,26,27,28,29,56,57,58]. In both cases, i.e., damaged by fretting wires and wires free of damage, the standard deviations calculated based on the obtained results were higher when no artificial aging was applied.

The calculated average values were analyzed using ANOVA testing and the results are presented in Table 4. Based on the calculated *p*-values, it can be determined that when the samples were not subjected to fretting damage, the values showed much more difference than when the wires were subjected to fretting with a 50 N load. Even though the calculated average values were not significantly different statistically, the influence of the heat treatment was noticeable.

Technological progress in the field of conductors includes both the proper selection of alloys with increased strength, corrosion resistance, and susceptibility to heat treatment, as well as the use of protective coatings to reduce friction, and thus fretting damage [31,36]. Currently, AlMgSi alloys are the most common material for the construction of overhead conductors. However, it is necessary to provide research on these alloys and search for new alloys or composite alternatives with improved properties [15,59]. A significant direction of development is the prediction of conductors’ durability using FEM, artificial intelligence, and machine learning algorithms. That, however, requires implementing experimental research results, making it possible to determine safe operating times, predict failures, and plan service for existing power grids more accurately.

## 5. Conclusions

The current study considered the influence of fretting damage on the fatigue strength of AA6101 alloy wires. Artificial aging was implemented to reduce this influence.

By implementing artificial aging, the mass decrease of wires due to fretting was reduced. The more cycles of fretting were applied, the better the results. In the case of 10,000 fretting cycles, the difference in mass decrease was more than 10% in favor of artificially aged wires.

Experimental research results provided the basis for creating a model of relations between the technology of obtaining AlMgSi wires and their operational properties. The resistance to fatigue failure accelerated by the fretting damage was in this case the main determinant of the operational properties. The fatigue resistance of wires damaged by fretting decreased significantly in all the tested cases in comparison to non-damaged wires. However, wires subjected to artificial aging at 160 °C for 9 h were affected the least, which was especially noticeable in the case of lower stress amplitudes. Since longer heat treatment times lead to overaging and thus decrease in the mechanical properties, 9 h is the proposed optimal aging time at this temperature for the purposes of increasing resistance to fretting fatigue.

Conductors are subjected to constant exposure to aeolian vibrations and thus fretting damage. Therefore, the fatigue strength of overhead line conductors should be tested on wires subjected to fretting damage. In the current study, it was proven experimentally that the fatigue strength of the damaged wires, and the service life of the conductor, increased when wires were artificially aged in comparison to non-artificially aged samples.

## Figures and Tables

**Table 1:** Deflection arrow and corresponding values of bending stress of round wires; applied to gauge length of 400 mm and diameter of 3.12 mm [20].

	Unit	Corresponding Values					
Deflection arrow	mm	125	112.5	100	87.5	75	
Amplitude stress	MPa	193	174	150	134	107	

**Table 2:** Chemical composition analysis in wt. %.

Element	Al	Mg	Si	Fe	Cu	Ti	Zn	Mn	Cr	V	B
Nominal [47]	Min. 97.8	0.4–0.9	0.3–0.7	Max. 0.4	Max. 0.05	Max. each 0.03; max. total 0.1					
Measured	98.66	0.57	0.58	0.19	0.001	0.002	0.002	0.002	0.001	0.004	0.009

**Table 3:** ANOVA analysis of the calculated means of mass decrease.

	Source of Variation	SS	df	MS	F	*p*-Value	F Crit
2 N	Between Groups	0.000036421	2	0.0000182	1.479438	0.25418	3.554557
	Within Groups	0.000221563	18	0.0000123			
	Total	0.000257984	20				
50 N	Between Groups	0.000017172	2	0.0000086	0.029176	0.971292	3.554557
	Within Groups	0.005297260	18	0.0002943			
	Total	0.005314432	20				

**Table 4:** ANOVA analysis of the calculated means of fatigue strength.

	Source of Variation	SS	df	MS	F	*p*-Value	F Crit
No fretting	Between Groups	647,575,506,551,821	2	323,787,753,275,911	1.006145	0.394487	3.885294
	Within Groups	3,861,722,566,703,720	12	321,810,213,891,976			
	Total	4,509,298,073,255,540	14				
Fretting damage 50 N	Between Groups	8,366,706,840	2	4,183,353,420	0.100261	0.905351	3.885294
	Within Groups	500,696,114,340	12	41,724,676,195			
	Total	509,062,821,180	14				

## Data Availability

The original contributions presented in the study are included in the article material; further inquiries can be directed to the corresponding author.
